# McSwan type V appendiceal intussusception and acute appendicitis coexistence in a 4-year-old child: a case report and review of the literature

**DOI:** 10.1093/jscr/rjaf236

**Published:** 2025-04-23

**Authors:** Reem Shihab, Hala O Abdallah, Ansam Nafaa, Yazan N Abdallah, Mahdi Kittaneh

**Affiliations:** Department of Medicine, Faculty of Medicine and Health Sciences, An-Najah National University, Rafedia Street, Nablus P400, State of Palestine; Department of Medicine, Faculty of Medicine and Health Sciences, An-Najah National University, Rafedia Street, Nablus P400, State of Palestine; Department of Medicine, Faculty of Medicine and Health Sciences, An-Najah National University, Rafedia Street, Nablus P400, State of Palestine; Department of Surgery, Rafedia Hospital, Rafedia Street, Nablus P400, State of Palestine; Department of Surgery, Rafedia Hospital, Rafedia Street, Nablus P400, State of Palestine

**Keywords:** appendicitis, appendiceal intussusception, McSwan type V, pediatrics

## Abstract

Appendicitis, an inflammation of the appendix, and intussusception, the invagination of one intestinal segment into a distal segment, both have overlapping presentations, which can pose diagnostic and therapeutic challenges. This report describes a 4-year-old girl with coexisting McSwain type V appendiceal intussusception and appendicitis. This rare case highlights appendicitis as a potential lead point for intussusception.

## Introduction

Appendicitis is characterized by the inflammation of the vermiform appendix [1]. With an incidence of 5.7–57 per 100 000 [2], appendicitis is the leading cause of acute abdomen [1], the most common indication for emergency abdominal surgery worldwide, and a frequent reason for emergency department consultations [3]. The clinical presentation of appendicitis varies with age. In adults, right lower quadrant (RLQ) pain, abdominal rigidity, and periumbilical pain radiating to the RLQ are key signs. In pediatrics, absent or diminished bowel sounds, positive psoas, obturator, and Rovsing signs are more reliable. Scoring systems such as the Alvarado score and Pediatric Appendicitis Score aid diagnosis, and ultrasonography is the recommended first-line imaging [4]. Appendectomy is the standard treatment for appendicitis across all age groups [5, 6], though conservative management of uncomplicated cases requires more evidence [1].

Intussusception, a common cause of intestinal obstruction in children, has an incidence of 31.61 per 100 000 in children under 5 years. It is defined as the invagination of one intestinal segment into another, commonly the ileum into the cecum [6–8]. Most cases (90%) are idiopathic, while 5% involve pathological lead points such as Meckel’s diverticulum, lymphoid hyperplasia, or intestinal polyps. Clinical features include intermittent abdominal pain, palpable mass, bilious vomiting, ‘currant jelly’ stools, and altered mental status [9]. Untreated intussusception can result in ischemia, perforation, peritonitis, and death. Diagnosis and reduction are typically achieved with enemas, with a success rate exceeding 80%, while surgery is reserved for complications or failed reduction [10].

Although common individually, concurrent intussusception and appendicitis are extremely rare, with an incidence of 0.01% [11]. This case report describes a 4-year-old girl with appendicitis and concurrent intussusception found intraoperatively, highlighting this rare combination and contributing to the limited knowledge to aid in future diagnosis and management.

## Case presentation

**Figure 1:**
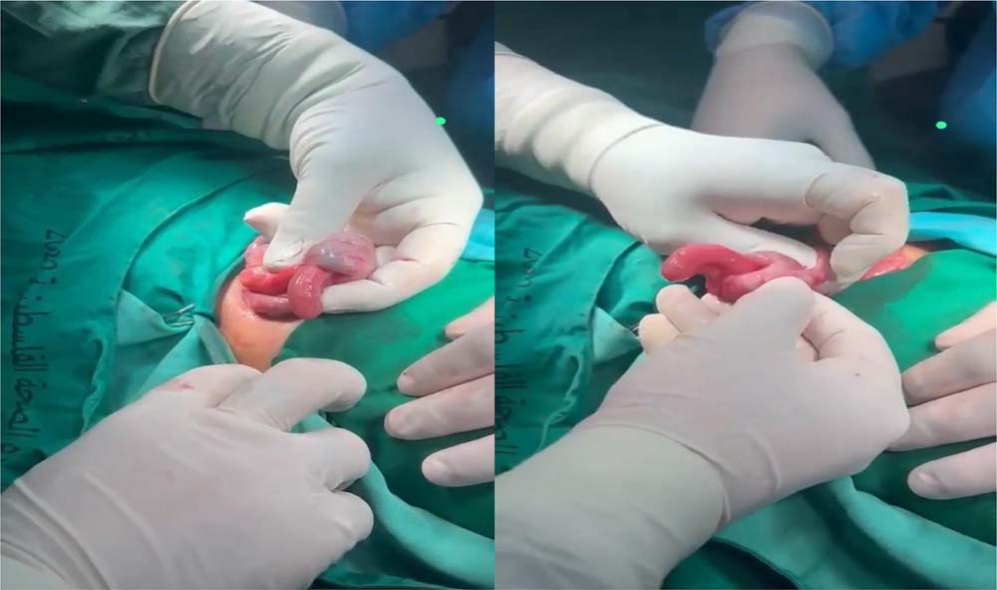
Ileocecal intussusception with the inflamed appendix invaginated inside the intussusception.

We report a 4-year-old girl presented to the emergency department at Tubas Turkish Hospital with persistent cramping right lower quadrant abdominal pain that started one day ago. The pain began in the periumbilical region and then shifted to the RLQ, associated with loss of appetite and one episode of non-bilious vomiting, but with no diarrhea or bloody stool. The child appeared ill, but her vital signs were stable, except for the body temperature which was 38.2°C. The abdomen was soft, not distended, with severe tenderness in the RLQ. Palpation of the left lower quadrant elicited pain in the RLQ. Bowel sounds were normal. Then the patient was referred to a hospital for further evaluation and treatment. Laboratory tests revealed a C-reactive protein level of 10.81 mg/L (normal reference range in our hospital laboratory: 0–5 mg/L) and other lab values were in the normal range. Ultrasonography of the abdomen was done and revealed a 2.8 × 2.1 cm target sign in the right upper quadrant with a fat core inside and a few enlarged, inflammatory-looking lymph nodes. There was also an adjacent tubular, blind-ended, non-compressible structure measuring ~1.2 cm, surrounded by edematous fat planes, suggesting ileocecal intussusception with acute appendicitis. Additionally, multiple diffuse enlarged lymph nodes up to 1.1 cm with inflammatory characteristics were noted. A small rim of pelvic free fluid was observed, and other organs appeared normal. Urgent surgery was performed under general anesthesia, using a Lanz incision. During the procedure, the cecum was identified, revealing ileocecal intussusception with the inflamed appendix invaginated inside the intussusception (Fig. 1).

Manual reduction and appendectomy were successfully done, and the pathologic specimen confirmed acute appendicitis which is measured 7 × 1 × 1 cm.

The perioperative period was uneventful and the patient discharged on Day 6 post operation, with normal physical finding and no complications.

## Discussion

We report a rare case of a 4-year-old girl with acute appendicitis co-existing with intussusception. The patient presented with symptoms typical of acute appendicitis, including abdominal pain and vomiting. Abdominal ultrasonography revealed the target sign and a blind-ended, non-compressible structure surrounded by edematous fat, leading to a diagnosis of ileocecal intussusception with acute appendicitis. Intraoperatively, the inflamed appendix was found between normal ileal loops. Manual reduction of the viable terminal ileum and appendectomy were successfully performed.

Appendiceal inflammation is the most common cause of appendiceal intussusception in pediatrics [11]. Although histological findings confirmed acute appendicitis, it remains unclear whether the appendicitis caused the intussusception or vice versa. Without imaging, misdiagnosis could have led to delayed treatment, bowel necrosis, and the need for bowel resection. This highlights the critical role of early imaging in identifying coexisting conditions and ensuring timely surgical intervention.

McSwain’s classification system defines five types of appendiceal intussusception, with our case classified as Type V: complete inversion of the appendix into the cecum [12]. A retrospective review of 210 pediatric appendicitis cases reported abdominal pain in 99%, vomiting in 80%, and fever in 61.9%, all of which were present in our patient except for diarrhea [13]. Similarly, a review of intussusception cases showed abdominal pain in 100% and vomiting in 91% [14], underscoring the diagnostic challenge posed by overlapping symptoms.

A PubMed search (2000–2024) for pediatric cases of appendicitis with intussusception identified 12 studies and 13 cases, all case reports. The mean age was 5 years, with 63.63% male patients. Abdominal pain was the most common symptom (72.7%), followed by vomiting (54.5%) and fever (18.18%). Abdominal tenderness was noted in 81% of cases [10].

This case contributes to the limited literature on this rare condition. It emphasizes the importance of considering coexisting appendicitis and intussusception in the differential diagnosis, particularly in cases with overlapping symptoms, to minimize misdiagnosis and improve patient outcomes.

## Data Availability

We obtained permission from the patient's family to use all the materials for this case report and all materials used belong to the archive of the hospital in this case report.
